# 2538: Artificial urinary sphincter (AUS) placement after failed urethral sling: Impact of sling removal and proximal cuff placement

**DOI:** 10.1017/cts.2017.292

**Published:** 2018-05-10

**Authors:** Arnav Srivastava, Gregory Joice, Madeline Manka, Nikolai Sopko, Edward Wright

**Affiliations:** Johns Hopkins University School of Medicine, Baltimore, MD, USA

## Abstract

OBJECTIVES/SPECIFIC AIMS: Perineal urethral sling placement is an option for men with mild to moderate post-prostatectomy stress urinary incontinence (SUI). However, men with persistent incontinence after sling placement often require secondary artificial urinary sphincter (AUS) placement, made difficult by the sling occupying the proximal bulbar urethra. This proximal section has a thicker corpus spongiosum which may mitigate cuff-induced ischemia and subsequent urethral atrophy. The authors report a series of AUS placements after failed sling, using sling revision or removal to access the proximal urethra. METHODS/STUDY POPULATION: Cutting the sling arms during urethral cuff placement increased urethral exposure and mobility. If feasible, completely removing the sling allowed the most proximal cuff site; but if dissection was felt unsafe, the mesh was left in situ and the cuff placed distally. This study is a retrospective cohort design of patients with SUI who underwent AUS placement after failed sling from 2010 to 2016. Variables included baseline patient characteristics, SUI severity, intraoperative variables, and postoperative outcomes. AUS failure, defined as infection, erosion or urethral atrophy, was analyzed at 12 and 96 months using univariate and multivariable logistic regression. RESULTS/ANTICIPATED RESULTS: Over the study period, 29 patients underwent AUS placement after failed sling. At the time of AUS placement, mean urethral circumference was 6.2 cm and 68% of patients had a 4.5 cm cuff placed; no cases required a 3.5 cm cuff. Seventy-three percent of cases were after transobturator sling placement (27% bone-anchored) and 45% of slings were explanted. AUS failure rate at 12 and 96 months was 17.8% and 45%, respectively; atrophy was the most common indication. Prior transobturator sling placement had lower rates of both 12 month (9.1% vs. 57%, *p*=0.006) and 96 month (36% vs. 71%, *p*=0.11) failure, though the latter was not statistically significant. Sling explant was not a significant predictor of 12 month (*p*=0.12) or 96 month failure (*p*=0.17). DISCUSSION/SIGNIFICANCE OF IMPACT: Sling revision during AUS placement helps expose the wider proximal urethra, allowing larger cuff size placement. This procedure appears safe, with low rates of erosion and short-term failure—albeit with high rates of long-term urethral atrophy possibly due to more significant dissection causing devascularization. However, sling removal was not a significant predictor of failure. The transobturator sling’s smaller profile may result in less trauma to urethra—possibly explaining the improved outcomes.

